# Contributions to the knowledge of oribatid mites of Indonesia. 1. The genus *Allogalumna* (Galumnidae) with descriptions of two new species (Acari, Oribatida)

**DOI:** 10.3897/zookeys.529.6326

**Published:** 2015-10-26

**Authors:** Sergey G. Ermilov, Dorothee Sandmann, Bernhard Klarner, Rahaju Widyastuti, Stefan Scheu

**Affiliations:** 1 Tyumen State University, Tyumen, Russia; 2 Georg August University Göttingen, J.F. Blumenbach Institute of Zoology and Anthropology, Göttingen, Germany; 3 Institut Pertanian Bogor, Bogor, Indonesia

**Keywords:** Oribatid mites, *Allogalumna*, new species, new record, Indonesia

## Abstract

Two new species of oribatid mites of the genus *Allogalumna* (Oribatida, Galumnidae) are described from litter and soil materials of Sumatra, Indonesia. *Allogalumna indonesiensis* **sp. n.** is morphologically most similar to *Allogalumna borhidii* Balogh & Mahunka, 1979, *Allogalumna quadrimaculata* (Mahunka, 1988), *Allogalumna rotundiceps* Aoki, 1996 and *Allogalumna plowmanae* Balogh & Balogh, 1983; however, the new species differs by having densely ciliate bothridial heads, larger body size and absence of a median pore. *Allogalumna paranovazealandica* **sp. n.** is morphologically most similar to *Allogalumna novazealandica* Hammer, 1968; however, the new species differs by the shorter body length and barbed and curving postero-laterad bothridial setae. The genus *Allogalumna* is recorded for the first time in the Indonesian fauna.

## Introduction

At present, the oribatid mite fauna (Acari, Oribatida) of Indonesia is poorly known (Sellnick 1925, 1930; Willmann 1929, 1932; Csiszár 1961; Balogh and Mahunka 1968; Mahunka 1977, 1989, 1990; Hammer 1979, 1981a, 1981b, 1982; Aoki et al. 1994; Niedbała 2007, 2008). This work is a part of a study on Indonesian oribatids and based on material which was collected in 2013 in the framework of the interdisciplinary project “Ecological and socioeconomic functions of tropical lowland rainforest transformation systems (Sumatra, Indonesia)”. Litter and soil samples were taken along a land use gradient including rainforest, jungle rubber, rubber and oil palm plantations in Jambi Province. For more details on the study region and experimental design see Barnes et al. (2014).

This paper includes the data on taxa of *Allogalumna* Grandjean, 1936 (Galumnidae). During taxonomic identification, two new species of this genus were found. The main goal of the paper is to describe and illustrate these species under the names *Allogalumna indonesiensis* sp. n. and *Allogalumna paranovazealandica* sp. n.

*Allogalumna* is a genus that was proposed by Grandjean (1936) with *Galumna alamellae* Jacot, 1935 as type species. Based on updated generic diagnosis (Ermilov et al. 2013a), it comprises more than 40^1^ species collectively having a cosmopolitan distribution; *Allogalumna* has not been reported before in the Indonesian fauna. An identification key to all known species of this genus was given by Akrami (2015), while additional keys to selective species were presented by Balogh and Balogh (2002) and Ermilov and Anichkin (2014).

## Materials and methods

Exact collection locality and habitat are given in the respective “Material examined” section for each new species.

Specimens were mounted in lactic acid on temporary cavity slides for measurement and illustration. The body length was measured in lateral view, from the tip of the rostrum to the posterior edge of the ventral plate. Notogastral width refers to the maximum width in dorsal aspect. Lengths of body setae were measured in lateral aspect. All body measurements are presented in micrometers. Formulae for leg setation are given in parentheses according to the sequence trochanter–femur–genu–tibia–tarsus (famulus included). Formulae for leg solenidia are given in square brackets according to the sequence genu–tibia–tarsus.

General terminology used in this paper follows that of Grandjean (summarized by Norton and Behan-Pelletier 2009).

Drawings were made with a camera lucida using a Carl Zeiss transmission light microscope “Axioskop-2 Plus”.

## Descriptions

### Allogalumna indonesiensis sp. n.

Taxon classificationAnimaliaOribatidaGalumnidae

http://zoobank.org/2E8C0C04-C670-4191-AA46-3134623A5D09

Figs 1 , 2 , 3–4 , 5–9

#### Diagnosis.

Body size: 282–298 × 215–232. Rostral, lamellar and interlamellar setae minute. Bothridial setae with unilaterally dilated, densely ciliate head. Anterior notogastral margin not developed. Four pairs of porose areas rounded. Median pore absent. Postanal porose area elongate oval.

#### Description.

*Measurements*. Body length: 282 (holotype: male), 282–298 (five paratypes: two females and three males); notogaster width: 215 (holotype), 215–232 (five paratypes). Without sexual dimorphism.

*Integument*. Body color brown. Body surface, pteromorphs, subcapitular mentum, genital and anal plates, and legs smooth.

**Figure 1.**
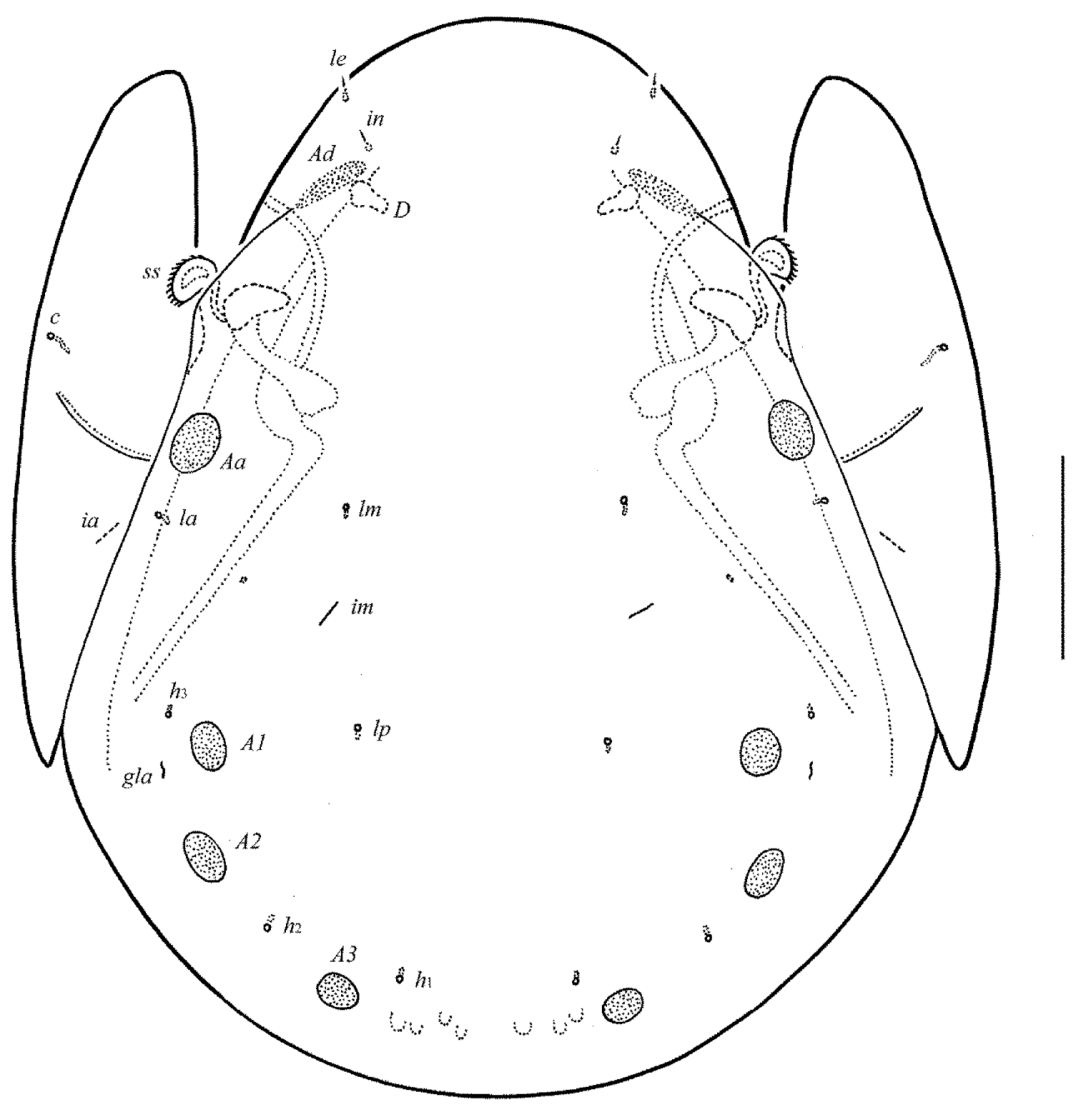
*Allogalumna indonesiensis* sp. n., adult: dorsal view. Scale bar 50 μm.

*Prodorsum* (Figs 1, 3, 5). Rostrum broadly rounded. Sublamellar lines (*S*) distinct, curving backwards. Rostral (*ro*), lamellar (*le*) and interlamellar (*in*) setae minute (all 4), thin, smooth. Bothridial setae (*bs*) comparatively short (49–53), with unilaterally dilated, densely ciliate head. Exobothridial setae and their alveoli absent. Porose areas *Ad* elongate oval, transversally oriented (16–20 × 6–8).

**Figure 2.**
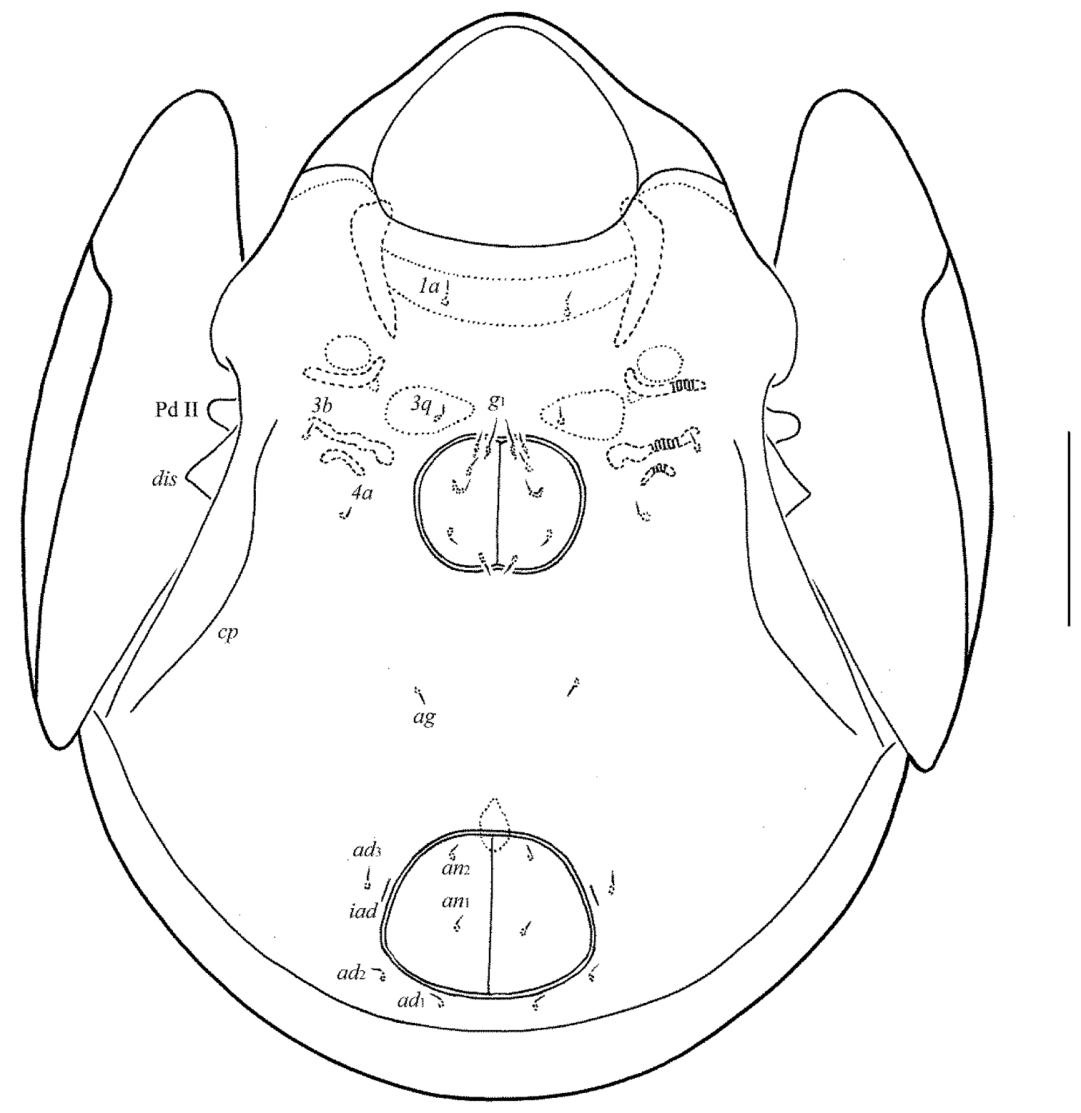
*Allogalumna indonesiensis* sp. n., adult: ventral view (gnathosoma and legs not shown). Scale bar 50 μm.

**Figures 3–4.**
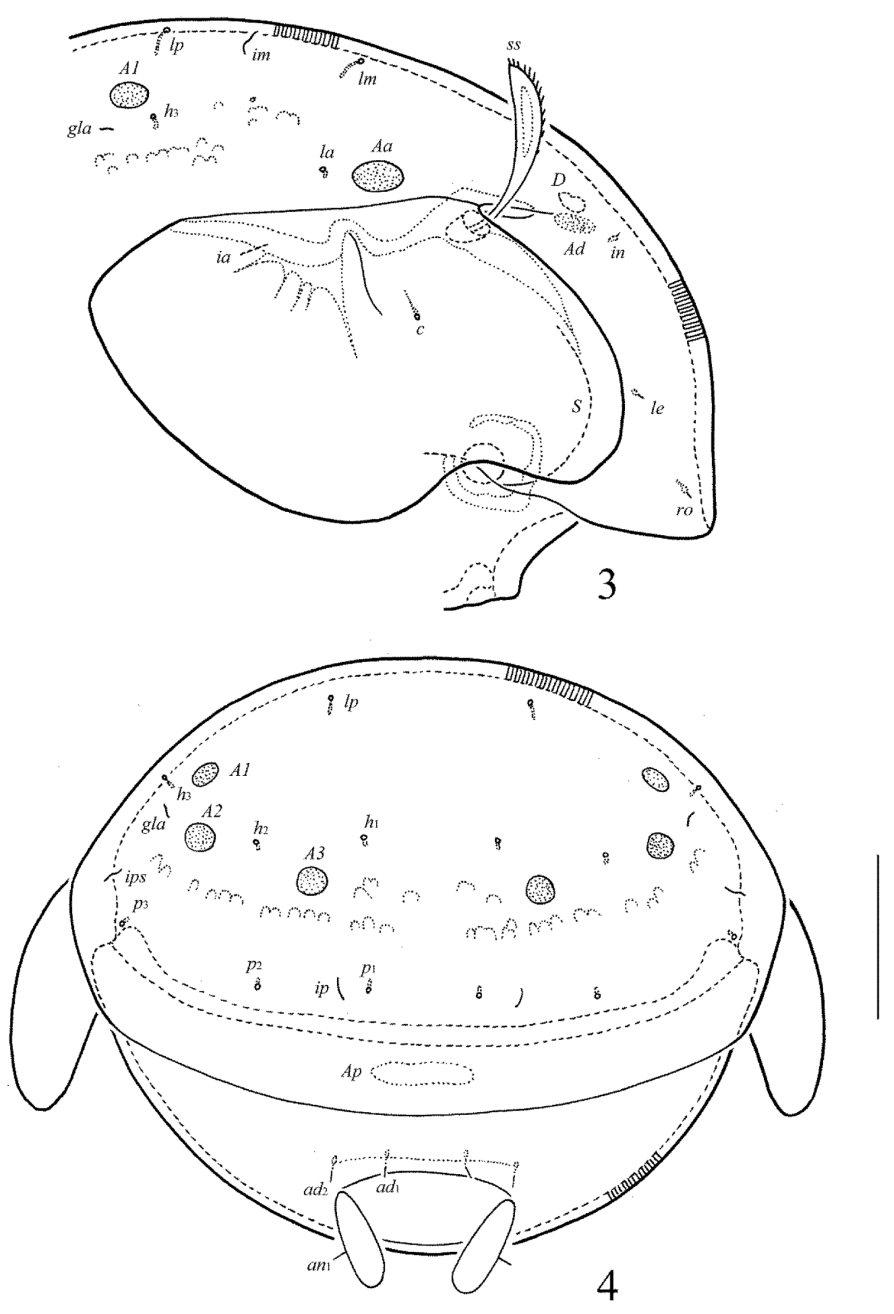
*Allogalumna indonesiensis* sp. n., adult: **3** anterior part of body, lateral view (gnathosoma and leg I not shown) **4** posterior view. Scale bar 50 μm.

**Figures 5–9.**
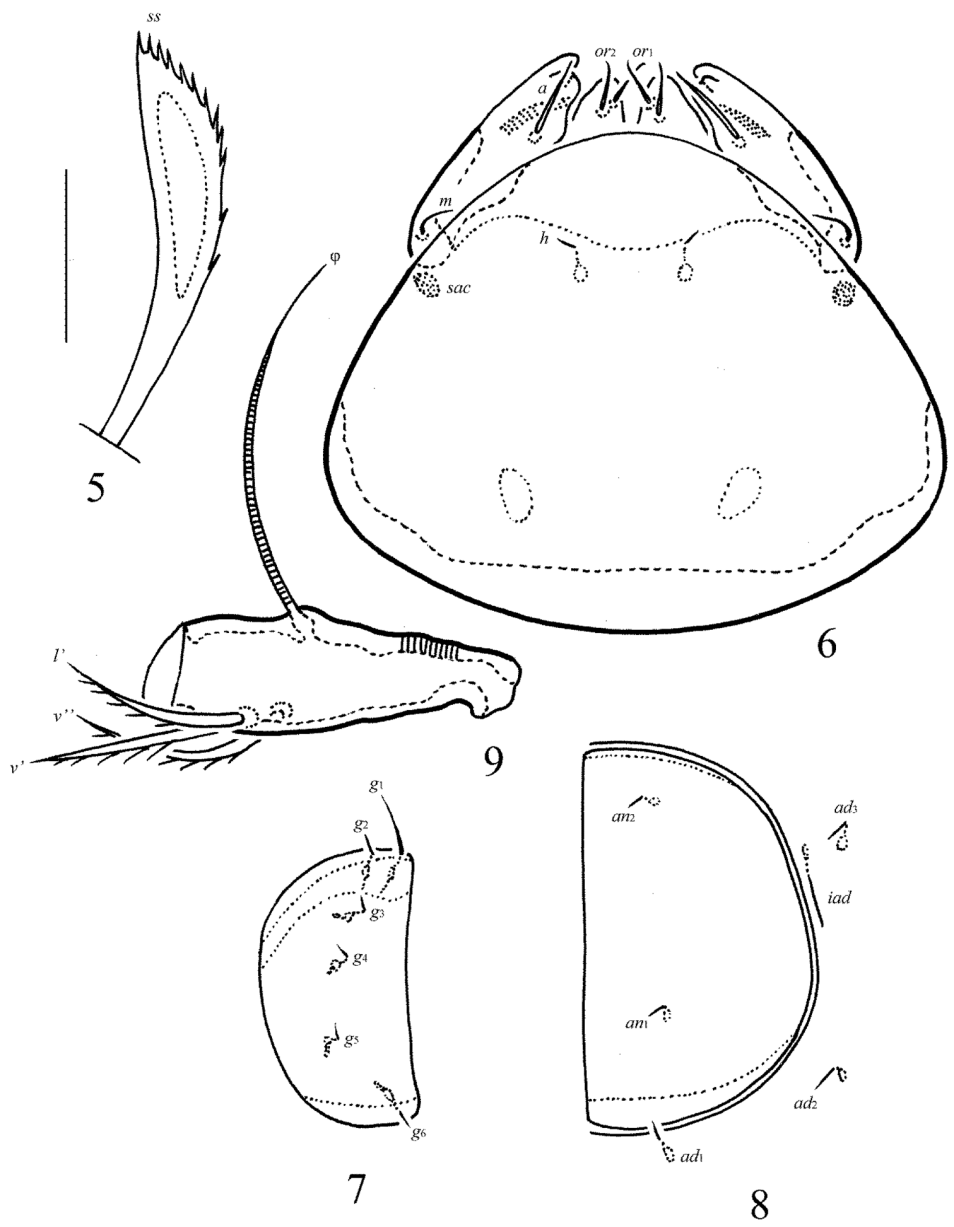
*Allogalumna indonesiensis* sp. n., adult: **5** bothridial seta **6** subcapitulum, ventral view **7** genital plate, right **8** anal plate, left, and adanal setae **9** tibia of leg IV, right, antiaxial view. Scale bar 20 μm.

*Notogaster* (Figs 1, 3, 4). Anterior notogastral margin not developed. Dorsophragmata (*D*) of medium size, elongated longitudinally. Notogastral setae represented by 10 pairs of alveoli. Four pairs of porose areas rounded, with distinct margins: *Aa* (16–18) usually slightly larger than *A1*, *A2* and *A3* (all 12–16). Setal alveoli *la* inserted posteriorly to *Aa*. Median pore absent in males and females. All lyrifissures (*ia*, *im*, *ip*, *ih*, *ips*) distinct, *im* located between setal alveoli *lm* and *lp*. Opisthonotal gland openings (*gla*) located laterally to *A1*.

*Gnathosoma* (Fig. 6). Morphology of subcapitulum, palps and chelicerae typical for most Galumnidae (for example, see Engelbrecht 1969, 1972; Ermilov and Anichkin 2010, 2011; Ermilov et al. 2011, 2013b; Bayartogtokh and Akrami 2014). Subcapitulum size: 61–69 × 61–69. Subcapitular setae setiform, smooth, *a* (10–12) longer than *m* (6–8) and *h* (4), *a* thickest, *h* thinnest. Two pairs of adoral setae (*or*_1_, *or*_2_, 8) thin, indistinctly barbed. Palps (57) with typical setation: 0–2–1–3–9(+ω). Axillary sacculi (*sac*) distinct. Chelicerae (77) with two setiform, barbed setae; *cha* (28) longer than *chb* (16). Trägårdh’s organ long, tapered.

*Epimeral and lateral podosomal regions* (Fig. 2). Anterior tectum of epimere I smooth. Apodemes 1, 2, sejugal and 3 well visible. Four pairs of short (all 4), thin setae, setal formula: 1–0–1–2. Pedotecta II (Pd II) scale-like in lateral view, rounded distally in ventral view. Discidia (*dis*) sharply triangular. Circumpedal carinae (*cp*) distinct, directed slightly laterally to setae *3b*.

*Anogenital region* (Figs 2, 4, 7, 8). Six pairs of genital (*g*_1_, 8; *g*_2_–*g*_6_, 4), one pair of aggenital (*ag*, 4), two pairs of anal (*an*_1_, *an*_2_, 4) and three pairs of adanal (*ad*_1_–*ad*_3_, 4) setae thin, smooth. Two genital setae on anterior edge of each genital plate. Adanal setae *ad*_3 _inserted laterally to adanal lyrifissures (*iad*). Postanal porose area (*Ap*) elongate oval, transversally oriented (28–32 × 6–8).

*Legs* (Fig. 9). Morphology of leg segments, setae and solenidia typical for most Galumnidae (for example, see Engelbrecht 1969, 1972; Ermilov and Anichkin 2010, 2011; Ermilov et al. 2011; Bayartogtokh and Akrami 2014). Tridactylous; claws smooth. Formulas of leg setation and solenidia: I (1–4–3–4–20) [1–2–2], II (1–4–3–4–15) [1–1–2], III (1–2–1–3–15) [1–1–0], IV (1–2–2–3–12) [0–1–0]; homology of setae and solenidia indicated in Table 1. Solenidion φ of tibiae IV inserted dorsally at about 2/3 length of segment.

**Table 1.** Leg setation and solenidia of adult *Allogalumna indonesiensis* sp. n. (same data for *Allogalumna paranovazealandica* sp. n.)

Leg	Tr	Fe	Ge	Ti	Ta
I	*v*’	*d*, ﻿(*l*), *bv*’’	(*l*), *v*’,﻿ ε	(*l*), ﻿(*v*),﻿ φ_1_, φ_2_	(*ft*), ﻿(*tc*), ﻿(*it*), ﻿(*p*), ﻿(*u*), ﻿(*a*), *s*, ﻿(*pv*), *v*’, ﻿(*pl*), *l*’’, ﻿ε,﻿ ω_1_, ω_2_
II	*v*’	*d*, ﻿(*l*), *bv*’’	(*l*), *v*’,﻿ σ	(*l*), ﻿(*v*),﻿ φ	(*ft*), ﻿(*tc*), ﻿(*it*), ﻿(*p*), ﻿(*u*), ﻿(*a*), *s*, ﻿(*pv*),﻿ ω_1_, ω_2_
III	*v*’	*d*, *ev*’	*l*’,﻿ σ	*l*’, ﻿(*v*),﻿ φ	(*ft*), ﻿(*tc*), ﻿(*it*), ﻿(*p*), ﻿(*u*), ﻿(*a*), *s*, ﻿(*pv*)
IV	*v*’	*d*, *ev*’	*d*, *l*’	*l*’, ﻿(*v*),﻿ φ	*ft*’’, ﻿(*tc*), ﻿(*p*), ﻿(*u*), ﻿(*a*), *s*, ﻿(*pv*)

Note: Roman letters refer to normal setae, Greek letters to solenidia (except ε = famulus). Single prime (‘) marks setae on the anterior and double prime (“) setae on the posterior side of a given leg segment. Parentheses refer to a pair of setae. Tr – trochanter , Fe – femur , Ge – genu , Ti – Tibia , Ta – tarsus .

#### Material examined.

Holotype (male): Indonesia, Sumatra, Harapan landscape, Jungle rubber agroforest, research site HJ2 (project site number), 01°49'31.9"S, 103°17'39.2"E, 84 m a.s.l., from forest floor litter material. Two paratypes (female and male): Indonesia, Sumatra, Bukit Duabelas landscape, secondary rainforest, research site BF1, 01°59'42.5"S, 102°45'08.1"E, 83 m a.s.l., from forest floor litter material. Three paratypes (female and two males): Indonesia, Sumatra, Bukit Duabelas landscape, Jungle rubber agroforest, research site BJ5, 02°08'35.6"S, E 102°51'04.7"E, 51 m a.s.l., from upper soil layer (0–5 cm). All specimens were collected by Bernhard Klarner (15.XI.2013) and determined and collected to morphospecies level by Dorothee Sandmann.

#### Type deposition.

The holotype is deposited in LIPI (Indonesian Institute of Science) Cibinong, Indonesia; three paratypes are deposited in the collection of the Senckenberg Museum, Görlitz, Germany; two paratypes are deposited in the collection of the Tyumen State University Museum of Zoology, Tyumen, Russia.

#### Etymology.

The specific name *indonesiensis* refers to the country of origin, Indonesia.

#### Remarks.

*Allogalumna indonesiensis* sp. n. is most similar to *Allogalumna borhidii* Balogh & Mahunka, 1979 from the Neotropical region (see Balogh and Mahunka 1979), *Allogalumna quadrimaculata* (Mahunka, 1988) from Malaysia (see Mahunka 1988), *Allogalumna rotundiceps* Aoki, 1996 from Japan and Vietnam (see Aoki 1996) and *Allogalumna plowmanae* Balogh & Balogh, 1983 from Australia (see Balogh and Balogh 1983) in having small body size, minute prodorsal setae, four pairs of rounded notogastral porose areas and bothridial setae with unilaterally dilated head. However, the new species differs from these species by having densely ciliate bothridial heads (versus slightly barbed in distal parts), larger body size (282–298 × 215–232 versus 243–264 × 193–202 in *Allogalumna borhidii*, 249 × 166^2^ in *Allogalumna quadrimaculata*, 212–219 × 155–160 in *Allogalumna rotundiceps* and 261 × 171 in *Allogalumna plowmanae*) and absence of a median pore (versus present in *Allogalumna borhidii*, *Allogalumna quadrimaculata* and *Allogalumna rotundiceps*).

### Allogalumna paranovazealandica sp. n.

Taxon classificationAnimaliaOribatidaGalumnidae

http://zoobank.org/5DB5344A-F409-47AF-AF3A-EBDBADD7F990

Figs 10 , 11 , 12–13 , 14–18

#### Diagnosis.

Body size: 282–298 × 199–215. Rostral, lamellar and interlamellar setae minute. Bothridial setae with unilaterally slightly dilated, elongated, barbed in medio-distal part head. Anterior notogastral margin not developed. Four pairs of porose areas rounded. Median pore present. Postanal porose area elongate oval.

#### Description.

*Measurements*. Body length: 282 (holotype: female), 282–298 (seven paratypes: two females and five males); notogaster width: 215 (holotype), 199–215 (seven paratypes). Without sexual dimorphism.

*Integument*. Body color brown. Body surface, pteromorphs, subcapitular mentum, genital and anal plates, and legs smooth.

**Figure 10.**
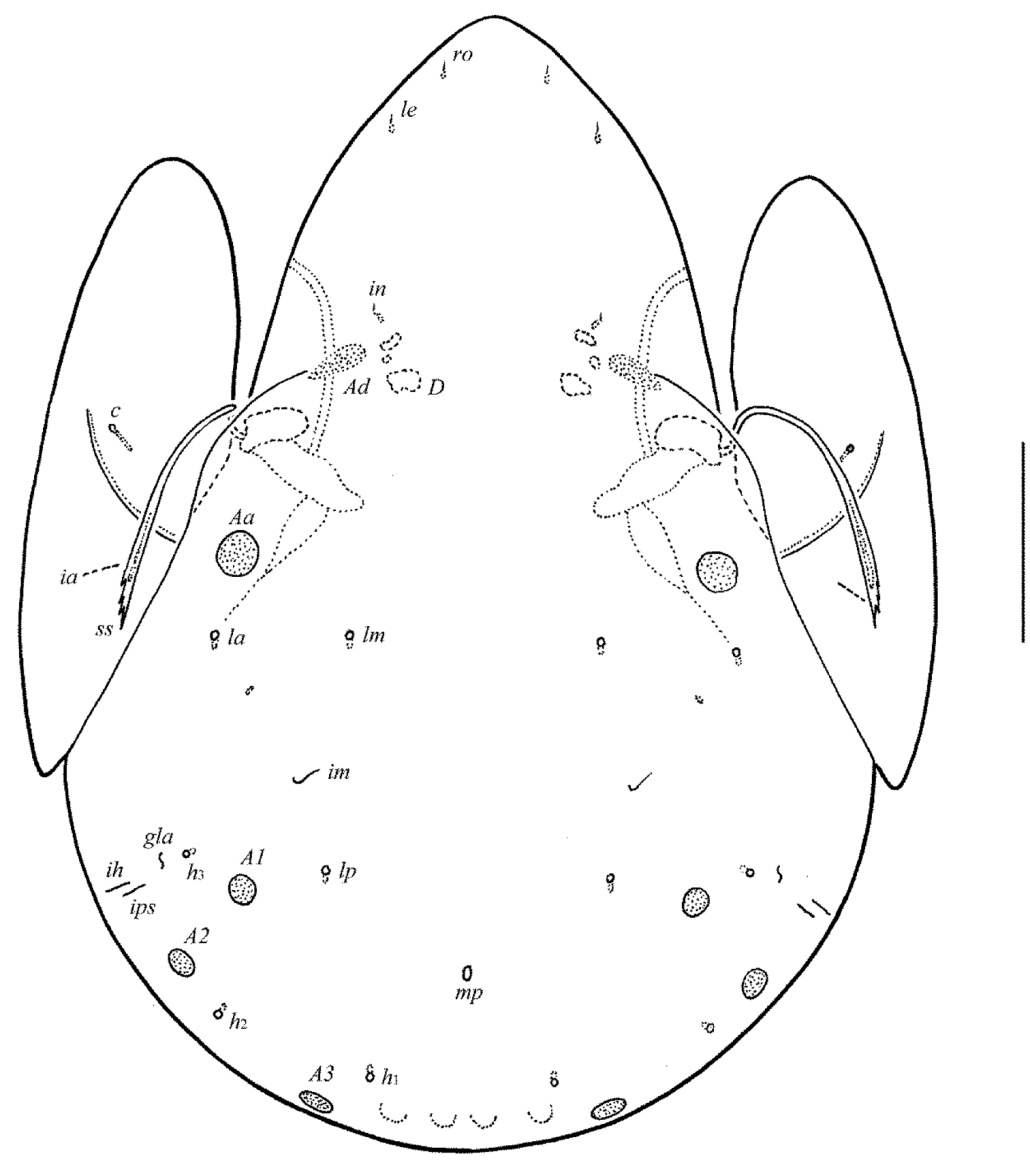
*Allogalumna paranovazealandica* sp. n., adult: dorsal view. Scale bar 50 μm.

*Prodorsum* (Figs 10, 12, 14). Rostrum broadly rounded. Sublamellar lines distinct, curving backwards. Rostral, lamellar and interlamellar setae minute (all 4), thin, smooth. Bothridial setae long (77–86), with unilaterally slightly dilated, elongated, barbed in medio-distal part head, directed postero-laterad. Exobothridial setae and their alveoli absent. Porose areas *Ad* elongate oval, transversally oriented (12–16 × 6–8).

**Figure 11.**
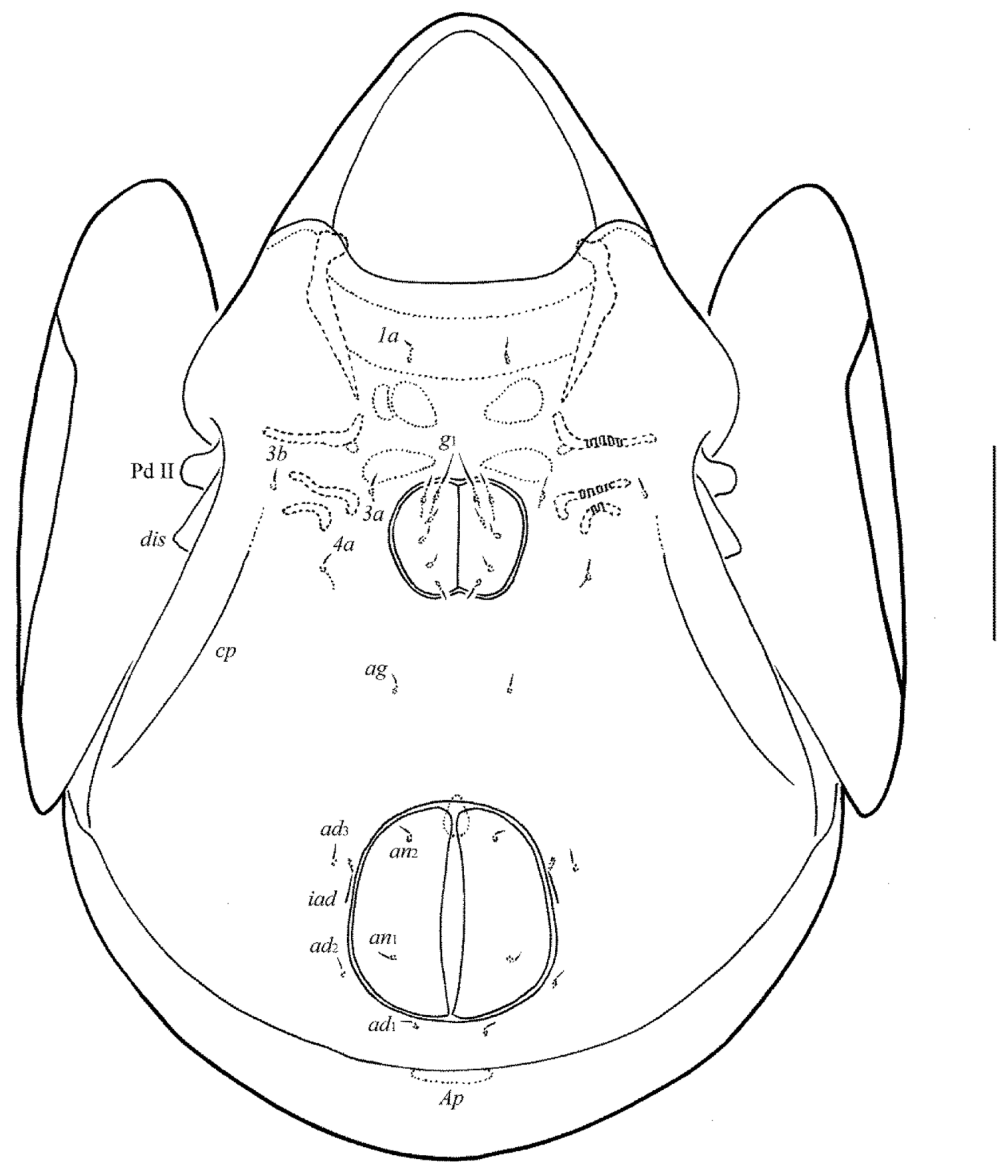
*Allogalumna paranovazealandica* sp. n., adult: ventral view (gnathosoma and legs not shown). Scale bar 50 μm.

**Figures 12–13.**
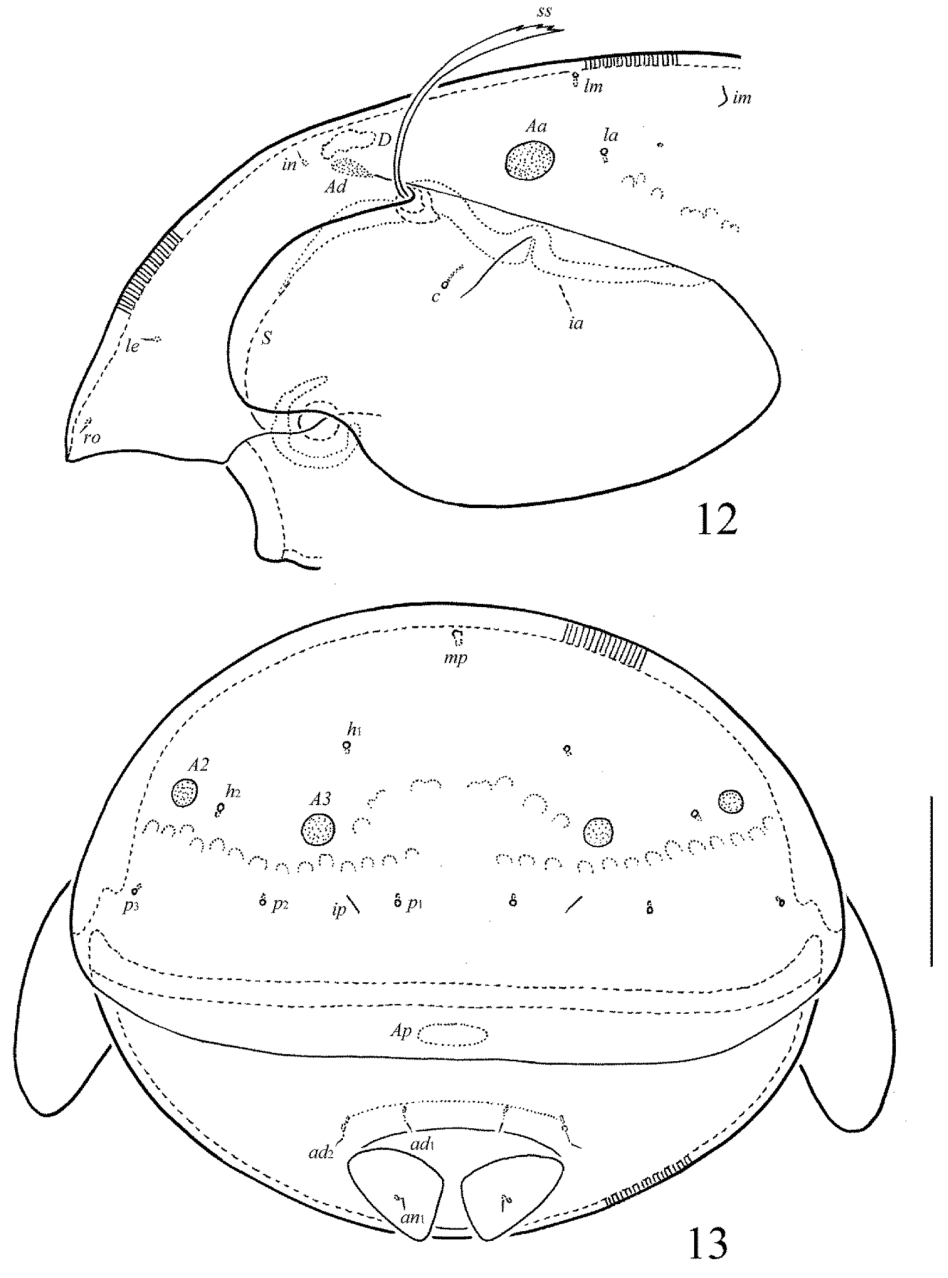
*Allogalumna paranovazealandica* sp. n., adult: **12** anterior part of body, lateral view (gnathosoma and leg I not shown) **13** posterior view. Scale bar 50 μm.

**Figures 14–18.**
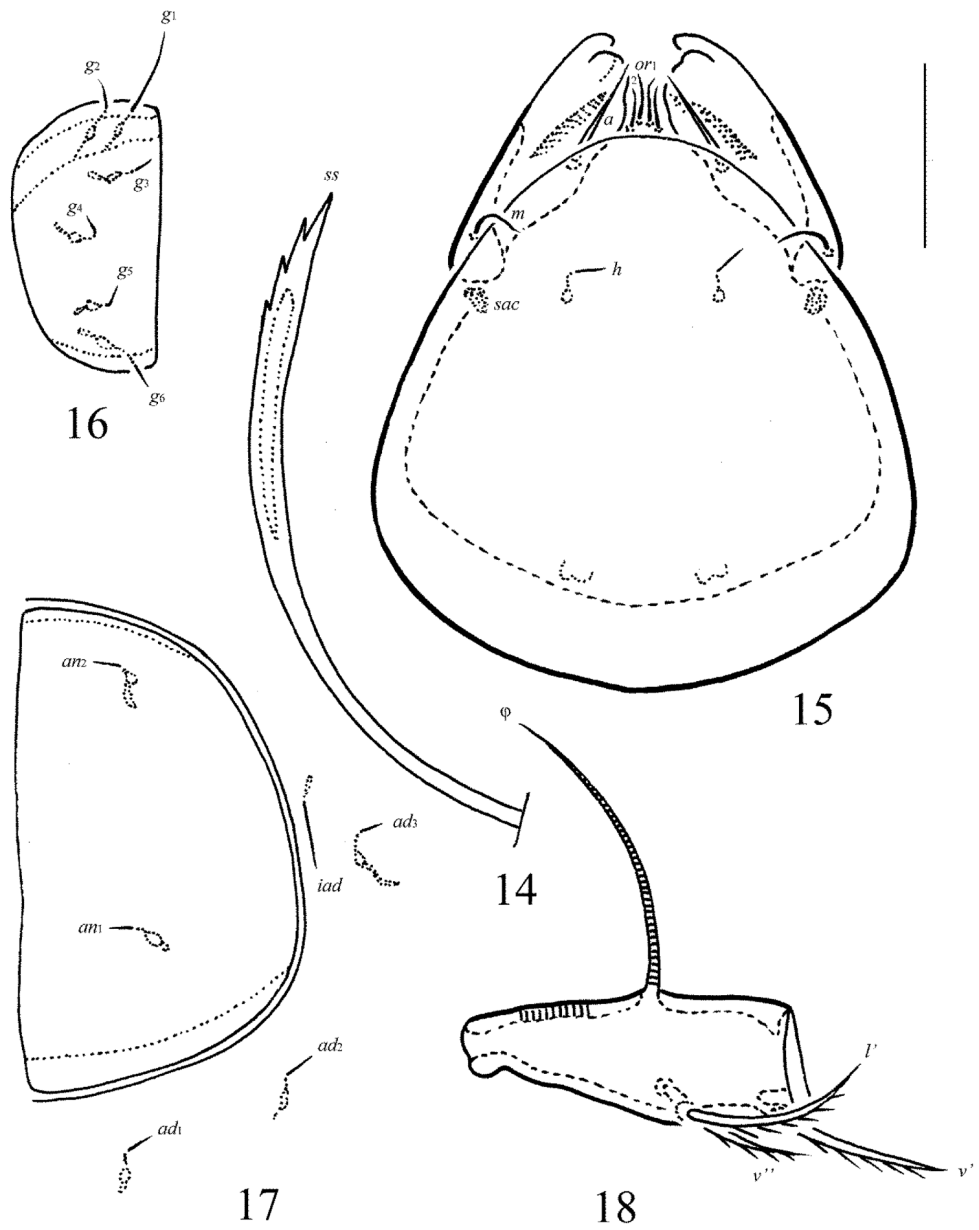
*Allogalumna paranovazealandica* sp. n., adult: **14** bothridial seta **15** subcapitulum, ventral view **16** genital plate, right **17** anal plate, left, and adanal setae **18** tibia of leg IV, left, antiaxial view. Scale bar 20 μm.

*Notogaster* (Figs 10, 12, 13). Anterior notogastral margin not developed. Dorsophragmata of medium size, elongated longitudinally. Notogastral setae represented by 10 pairs of alveoli. Four pairs of porose areas rounded, with distinct margins: *Aa* (14–16) larger than *A1*, *A2* and *A3* (all 6–10). Setal alveoli *la* inserted posteriorly to *Aa*. Median pore present in males and females, located between *A2*. All lyrifissures distinct, *im* located between setal alveoli *lm* and *lp.* Opisthonotal gland openings located laterally to *A1*.

*Gnathosoma* (Fig. 15). Morphology of subcapitulum, palps and chelicerae typical for most Galumnidae (for example, see Engelbrecht 1969, 1972; Ermilov and Anichkin 2010, 2011; Ermilov et al. 2011, 2013b; Bayartogtokh and Akrami 2014). Subcapitulum size: 73 × 61–65. Subcapitular setae setiform, smooth, *a* (12) longer than *m* (8) and *h* (4), *a* thickest, *h* thinnest. Two pairs of adoral setae (6–8) thin, indistinctly barbed. Palps (61) with typical setation: 0–2–1–3–9(+ω). Axillary sacculi (*sac*) distinct. Chelicerae (82) with two setiform, barbed setae; *cha* (28) longer than *chb* (16). Trägårdh’s organ long, tapered.

*Epimeral and lateral podosomal regions* (Fig. 11). Anterior tectum of epimere I smooth. Apodemes 1, 2, sejugal and 3 well visible. Four pairs of short (all 4), thin setae, setal formula: 1–0–1–2. Pedotecta II scale-like in lateral view, rounded distally in ventral view. Discidia sharply triangular. Circumpedal carinae indistinctly developed, directed to setae *3b*.

*Anogenital region* (Figs 11, 13, 16, 17). Six pairs of genital (*g*_1_, 10; *g*_2_–*g*_6_, 4), one pair of aggenital (4), two pairs of anal (4) and three pairs of adanal (4) setae thin, smooth. Two genital setae on anterior edge of each genital plate. Adanal setae *ad*_3 _inserted laterally to adanal lyrifissures. Postanal porose area elongate oval, transversally oriented (20 × 6–8).

*Legs* (Fig. 18). Morphology of leg segments, setae and solenidia typical for most Galumnidae (for example, see Engelbrecht 1969, 1972; Ermilov and Anichkin 2010, 2011; Ermilov et al. 2011; Bayartogtokh and Akrami 2014). Tridactylous; claws smooth. Formulas of leg setation and solenidia: I (1–4–3–4–20) [1–2–2], II (1–4–3–4–15) [1–1–2], III (1–2–1–3–15) [1–1–0], IV (1–2–2–3–12) [0–1–0]; homology of setae and solenidia indicated in c Solenidion φ of tibiae IV inserted dorsally at about 2/3 length of segment.

#### Material examined.

Holotype (female): Indonesia, Sumatra, Bukit Duabelas landscape, Jungle rubber agroforest, research site BJ5, 02°08'35.6"S, E 102°51'04.7"E, 51 m a.s.l., from upper soil layer (0–5 cm). Four paratypes (female and three males): Indonesia, Sumatra, Harapan landscape, Rubber plantation, research site HR2, 01°52'44.5"S, 103°16'28.4"E, 59 m a.s.l., from upper soil layer (0–5 cm). Three paratypes (female and two males): Indonesia, Sumatra, Harapan landscape, secondary rainforest, research site HF4, S 02°11'15.2"S, 103°20'33.4"E, from upper soil layer (0–5 cm). All specimens were collected by Bernhard Klarner (15.XI.2013) and determined and collected to morphospecies level by Dorothee Sandmann.

#### Type deposition.

The holotype is deposited in LIPI (Indonesian Institute of Science) Cibinong, Indonesia; three paratypes are deposited in the collection of the Senckenberg Museum, Görlitz, Germany; four paratypes are deposited in the collection of the Tyumen State University Museum of Zoology, Tyumen, Russia.

#### Etymology.

The specific name *paranovazealandica* refers to the morphological similarity of the new species to *Allogalumna novazealandica* Hammer, 1968.

#### Remarks.

*Allogalumna paranovazealandica* sp. n. is most similar to *Allogalumna novazealandica* Hammer, 1968 from New Zealand in having minute prodorsal setae, long bothridial setae with slightly dilated head, four pairs of rounded notogastral porose areas, median pore and elongated postanal porose area. However, the new species differs from the latter by the shorter body length (282–298 versus 400–410 in *Allogalumna novazealandica*) and barbed in medio-distal part and curving postero-laterad bothridial setae (versus smooth and straight, directed upwards-laterally in *Allogalumna novazealandica*).

## Supplementary Material

XML Treatment for Allogalumna indonesiensis

XML Treatment for Allogalumna paranovazealandica

## Footnotes

1 Subías (2004, updated 2015) included 37 species in *Allogalumna*.

2 Mahunka (1988) presented the following body size for *Allogalumna quadrimaculata* (systematic placement for this species in *Allogalumna* established by Ermilov and Bayartogtokh 2015): 389–405 × 275–300. We studied the sizes of two paratypes of *Allogalumna quadrimaculata*, and found 249 × 166. Hence, this corrected data could be used in future identification of *Allogalumna quadrimaculata*.

## References

[B1] Akrami MA (2015) A new species of *Allogalumna* (Acari, Oribatida, Galumnidae) from Iran, including a key to all species of the genus. Acta Zool Acad Sci Hung 63(1): 205–224. doi: 10.17109/AZH.61.3.1.2015

[B2] Aoki J (1996) Two new species of oribatid mites of the family Galumnidae from Okinawa Island. Edaphologia 56: 1–4.

[B3] Aoki J Takaku G Ito F (1994) Aribatidae, a new myrmecophilous oribatid mite family from Java. Int J Acarol 20: 3–10. doi: 10.1080/01647959408683994

[B4] Balogh J Balogh P (1983) New oribatid mites from Australia (Acari: Oribatei). Acta Zool Acad Sci Hung 29(1–3): 81–105.

[B5] Balogh J Balogh P (2002) Identification keys to the oribatid mites of the Extra-Holarctic regions. Vol. 1. Miskolc, Well-Press Publ Limited, 453 pp.

[B6] Balogh J Mahunka S (1968) New oribatids (Acari) from Indonesian soils. Opusc Zool Budapest 8(2): 341–346.

[B7] Balogh J Mahunka S (1979) New data to the knowledge of the oribatid fauna of the Neogaea (Acari). IV. Acta Zool Acad Sci Hung 25(1–2): 35–60.

[B8] Barnes AD Jochum M Mumme S Haneda NF Farajallah A Widarto TH Brose U (2014) Consequences of tropical land use for multitrophic biodiversity and ecosystem functioning. Nat Com 5: . doi: 10.1038/ncomms6351 10.1038/ncomms6351 PMC4220457 25350947

[B9] Bayartogtokh B Akrami MA (2014) The soil mite family Galumnidae of Iran (Acari: Oribatida). J Nat Hist 48(15–16): 881–917. doi: 10.1080/00222933.2013.840397

[B10] Csiszár MJ (1961) New oribatids from Indonesian soils (Acari). Acta Zool Acad Sci Hung 7(3–4): 345–366.

[B11] Engelbrecht CM (1969) Some South African species of the genus *Galumna* von Heyden, 1826 (Acari: Galumnidae). J Ent Soc South Afr 32(1): 99–122.

[B12] Engelbrecht CM (1972) Galumnids from South Africa (Galumnidae, Oribatei). Acarologia 14(1): 109–140.

[B13] Ermilov SG Anichkin AE (2010) Three new species of Galumnidae (Acari: Oribatida) from Cat Tien National Park, southern Vietnam. Zootaxa 2681: 20–34.

[B14] Ermilov SG Anichkin AE (2011) New oribatid mites of the genera *Pergalumna* and *Galumnella* (Acari, Oribatida, Galumnoidea) from Vietnam. Acarina 19(2): 242–251.

[B15] Ermilov SG Anichkin AE (2014) Two new species of oribatid mites of the family Galumnidae (Acari, Oribatida) from Vietnam. ZooKeys 382: 53–66. doi: 10.3897/zookeys.382.6831 24624019 10.3897/zookeys.382.6831 PMC3950421

[B16] Ermilov SG Bayartogtokh B (2015) Systematic placement of some taxa in the family Galumnidae (Acari, Oribatida). Zootaxa 3964(4): 489–493. doi: 10.11646/zootaxa.3964.4.8 26249459 10.11646/zootaxa.3964.4.8

[B17] Ermilov SG Sidorchuk EA Rybalov LB (2011) Three new species of oribatid mites (Acari: Oribatida: Galumnoidea) from Ethiopia. Int J Acarol 37 (Suppl. 1): 2–17. doi: 10.1080/01647954.2010.528799

[B18] Ermilov SG Starý J Sandmann D Marian F Maraun M (2013) New taxa and new records of oribatid mites of the family Galumnidae (Acari: Oribatida) from Ecuador. Zootaxa 3700(2): 259–270. doi: 10.11646/zootaxa.3700.2.4 26106726 10.11646/zootaxa.3700.2.4

[B19] Ermilov SG Weigmann G Tolstikov AV (2013b) Morphology of adult and juvenile instars of *Galumna obvia* (Acari, Oribatida, Galumnidae), with discussion of its taxonomic status. ZooKeys 357: 11–28. doi: 10.3897/zookeys.357.6404 24363576 10.3897/zookeys.357.6404 PMC3867166

[B20] Grandjean F (1936) Les Oribates de Jean Frédéric Hermann et de son pere. Ann Soc Ent France 105: 27–110.

[B21] Hammer M (1968) Investigations on the Oribatid fauna of New Zealand. Part III. Det Kong Dansk Vidensk Selsk Biol Skr 16(2): 1–96.

[B22] Hammer M (1979) Investigations on the oribatid fauna of Java. Det Kong Dansk Vidensk Selsk Biol Skr 22(9): 1–78.

[B23] Hammer M (1981a) On some oribatid mites from Java. Part I. Acarologia 22(1): 81–99.

[B24] Hammer M (1981b) On some oribatid mites from Java. Part II. Acarologia 22(2): 217–237.

[B25] Hammer M (1982) On a collection of oribatid mites from Bali, Indonesia (Acari: Cryptostigmata). Ent Scand 13: 445–464. doi: 10.1163/187631282X00291

[B26] Mahunka S (1977) Neue und interessante Milben aus dem Genfer Museum XX. Contribution to the oribatid fauna of S.E. Asia (Acari, Oribatida). Rev suisse Zool 84(1): 247–274. doi: 10.5962/bhl.part.91385 877511

[B27] Mahunka S (1988) New and interesting mites from the Geneva Museum LXI. Oribatids from Sabah (East Malaysia) III (Acari: Oribatida). Rev suisse Zool 95(3): 817–888. doi: 10.5962/bhl.part.81937 877511

[B28] Mahunka S (1989) New and interesting mites from the Geneva Museum LXV. Oribatids from Sumatra (Indonesia) I (Acari: Oribatida). Rev suisse Zool 96(3): 673–696. 877511

[B29] Mahunka S (1990) New and interesting mites from the Geneva Museum LXXI. New oribatids (Acari) from the Philippines and Indonesia. Arch Sci 43(3): 453–460.

[B30] Niedbała W (2007) New distributional records and redescriptions of oriental ptyctimous mites (Acari, Oribatida) of the Oriental region. Syst Appl Acarol 12: 73–79. doi: 10.11158/saa.12.1.9

[B31] Niedbała W (2008) New species of ptyctimous mites (Acari, Oribatida) from Borneo and Sumatra. Zootaxa 1786: 1–18.

[B32] Norton RA Behan-Pelletier VM (2009) Chapter 15. Oribatida. In: Krantz GW Walter DE (Eds) A Manual of Acarology. Texas Tech Univ Press, Lubbock, 430–564.

[B33] Sellnick M (1925) Javanische Oribatiden. Treubia 6: 459–475.

[B34] Sellnick M (1930) Zwei neue Oribatidengattungen aus Sumatra (Acar.). Zool Anz 86(9–10): 225–231.

[B35] Subías LS (2004) Listado sistemático, sinonímico y biogeográfico de los ácaros oribátidos (Acariformes: Oribatida) del mundo (excepto fósiles). Graellsia 60(número extraordinario): 3–305. Online version accessed in March 2015, 587 pp.

[B36] Willmann C (1929) Zwei neue Malaconothridae aus Java. Zool Anz 83(1–4): 89–92.

[B37] Willmann C (1932) Eine neue Sphaerozetes-Art aus Java (Oribatei, Acari). Zool Anz 99(5–6): 174–176.

